# Cambodia national health hotline - Participatory surveillance for early detection and response to disease outbreaks

**DOI:** 10.1016/j.lanwpc.2022.100584

**Published:** 2022-09-06

**Authors:** Channé Suy Lan, Samnang Sok, Kakada Chheang, Divon Mordechai Lan, Vuthy Soung, Nomita Divi, Sovann Ly, Mark Smolinski

**Affiliations:** a InSTEDD iLab Southeast Asia, Phnom Penh, Cambodia; b Communicable Disease Control Department, Ministry of Health, Cambodia; c School of Biological Sciences, University of Adelaide, Adelaide, Australia; d Telecom Cambodia, Phnom Penh, Cambodia; e Ending Pandemics, San Francisco, USA

## Introduction

In the assessment of global preparedness for the next pandemic, the least prepared countries are clustered in Central and West Africa and Southeast Asia.^1^ Although many developed countries have used participatory systems in the digital age to strengthen disease surveillance,^2^^,^^3^ the question arises whether Low and Middle Income Countries (LMICs) can take advantage of their increasing mobile network coverage to improve their disease surveillance and outbreak detection.

In this article we describe the case of Cambodia - how this Southeast Asian nation of 17 million people utilises mobile network connectivity for its participatory disease surveillance program. Rapid adaptation and fine-tuning of systems for COVID-19 response is also illustrated. We also discuss how other similar LMICs could benefit from Cambodia's COVID-19 experience and adapt telecommunication means to strengthen participatory surveillance and preparedness for the next outbreak.

The use of digital systems for disease surveillance has been in place in Cambodia for a number of years now. For example, the Cambodia Early Warning (CamEwarn) system, supported by WHO, monitors infectious diseases in the community and continues to have a significant impact. Although the CamEwarn is an important disease surveillance system used by Operational Districts (OD) upto to the national level, it does not scale down to the health centre level due to the lack of computers, Internet connectivity and technology know-how at many health centres. That left ODs with the task of manually collecting data from health centres using a mobile phone, pen and paper, and later entering it to the CamEwarn system.

In 2013 at the EpiHack Phnom Penh,^4^ an event that brought together software developers and global health experts, including the authors, we conceptualised a new Health Hotline system. Subsequently, the Hotline system was developed by InSTEDD iLab Southeast Asia (CL,KC) in close collaboration with the Communicable Disease Control Department (CCDC) of the Ministry of Health (SL,SS), in partnership with Telecom Cambodia (VS) and with the guidance and support of philanthropy (MS,ND). The Hotline-115 was launched in 2016, and was further enhanced with new capabilities for handling COVID contact tracing in 2020.

## The case of Cambodia's participatory surveillance “hotline-115”

Prior to 2016, Cambodia's infectious disease surveillance relied mainly on a weekly data collection - data was collected at the local health centres, by the Operational Districts (ODs), from which the reports were sent onwards to the Provincial Health Departments before finally reaching the Ministry of Health in Phnom Penh. The staff at the 87 ODs throughout the country were given US$5 in mobile phone credit each month, to use their personal mobile phones to call the health centres for the weekly information collection. They were tasked with calling all 1,236 health centres, to collect data related to 10 infectious diseases, provide information which included the number of cases and deaths, and were requested to enter the collected data to CamEwarn. As of 2015, the timeliness and completeness of the data collection from health centres was estimated to be 80%, and ODs spent a total of 65 hours per week on phone calls to collect the data from the health centres.

The new Hotline-115 system that launched in 2016^5^ was built as an extension to the CamEwarn system with multiple purposes of increasing the speed while reducing the cost of data collection from health centres and strengthening participatory surveillance and outbreak detection. The Hotline-115 provides a simple user interface and is available 24/7, and is provided toll-free with all mobile networks in the country.

**Figure 1:**
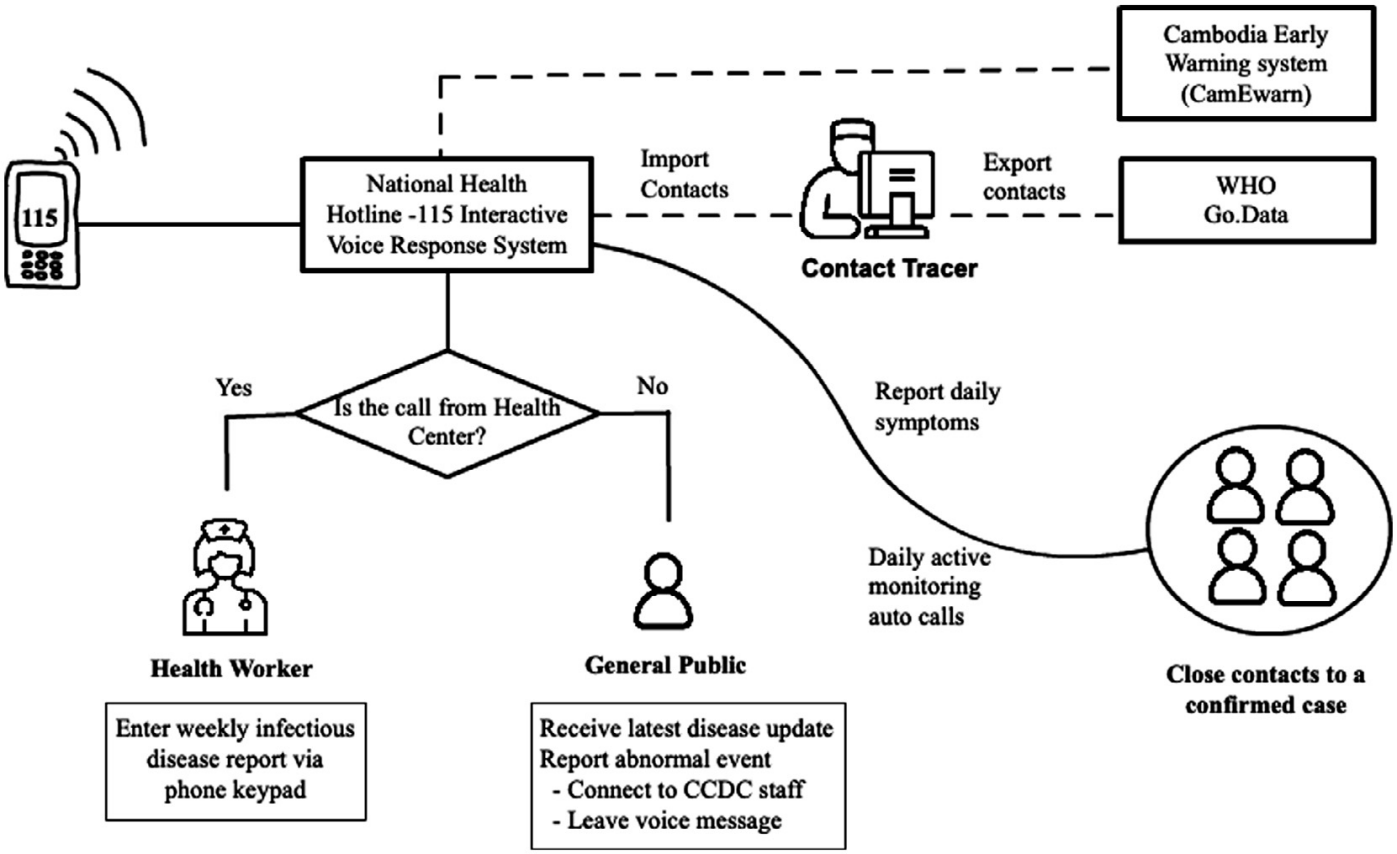
Diagram of the Hotline 115 system for data collection, public engagement and contact tracing.

The calls received by Hotline-115 are routed to the interactive voice response system (Figure 1). For incoming calls from a health centre, the system asks for the weekly report and health workers can then enter the number of cases and deaths using their phone keypad. The data is reviewed by the OD, then sent from the Hotline-115 system to CamEwarn. For calls received from the public, the system routes the caller to receive the latest disease update from CCDC. The caller may also connect to CCDC staff or leave a voice message to report any suspected cases of disease in their community.

## Results

The first breakthrough success of the system occurred on 20th January 2017, when the Hotline-115 received a call from a farmer in Svay Rieng, a rural province located in the southeastern corner of the country. The farmer reported what he thought might be an H5N1 case, several of which had previously occurred in the same province.^6^ The Ministry of Health immediately deployed a Rapid Response Team to investigate, and indeed the suspected case was confirmed, and the outbreak was contained.

In early 2020, when COVID-19 began spreading in Cambodia and supply of COVID-19 test kits was scarce, CCDC used Hotline-115 for screening testing requests from the public. Shortly after, the system was enhanced to conduct partially automated contact tracing. A contact tracer could export the list of close contacts from the WHO's Go.Data case recording system and import it to the Hotline-115 to perform automated calls in four languages to close contacts of confirmed cases, which repeat for 14 consecutive days and ask about symptoms. When the person receiving the automated call by Hotline-115 confirmed symptoms, the case was escalated to the contact tracing team which then followed up with a human call.

**Figure 2:**
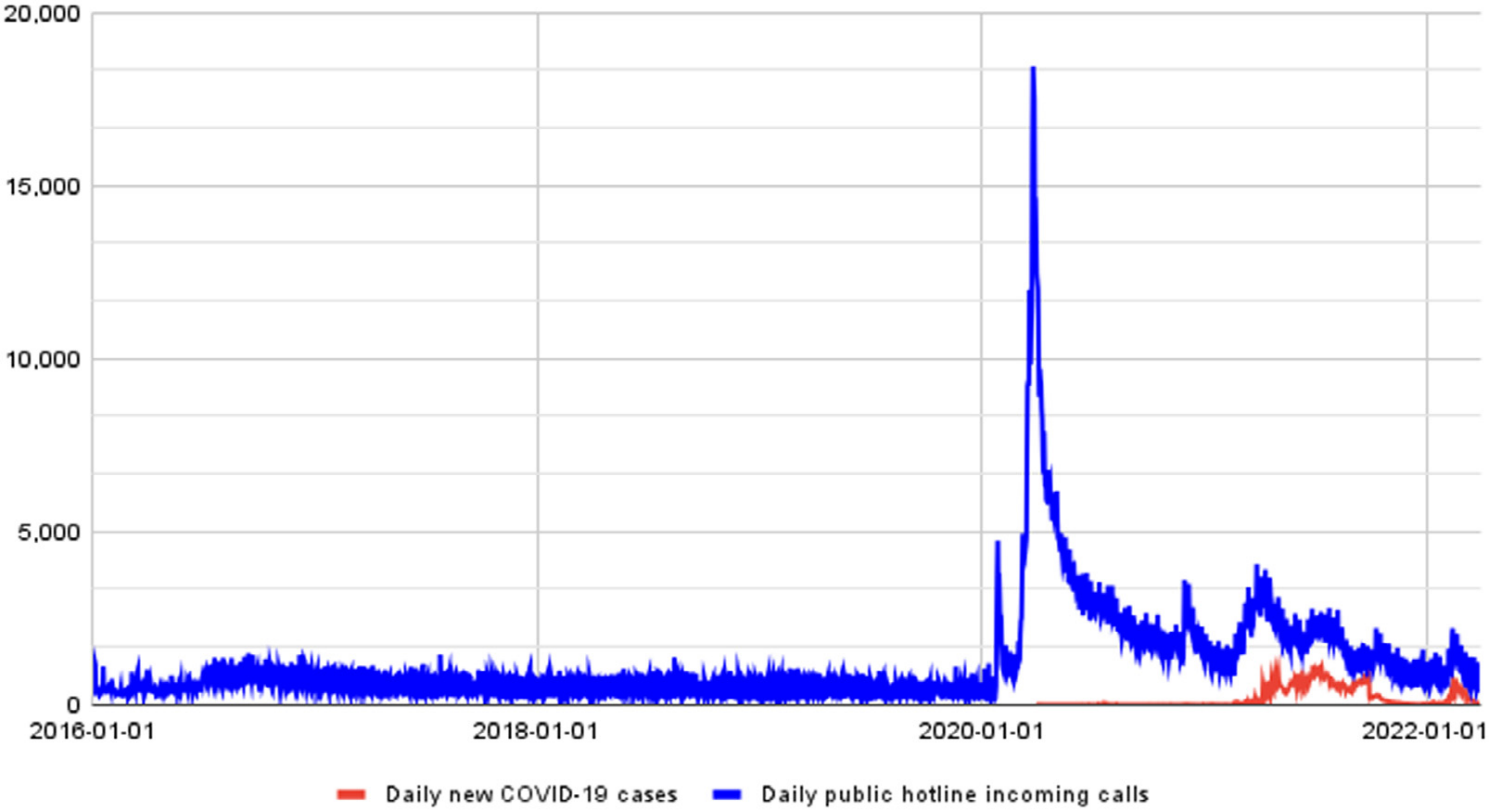
The incoming call volume to the Hotline 115 (blue line, source: Hotline-115 system logs) and the number of confirmed COVID-19 cases in the country (red line, source: CCDC^7^) between January 2016 and March 2022.

**Figure 3:**
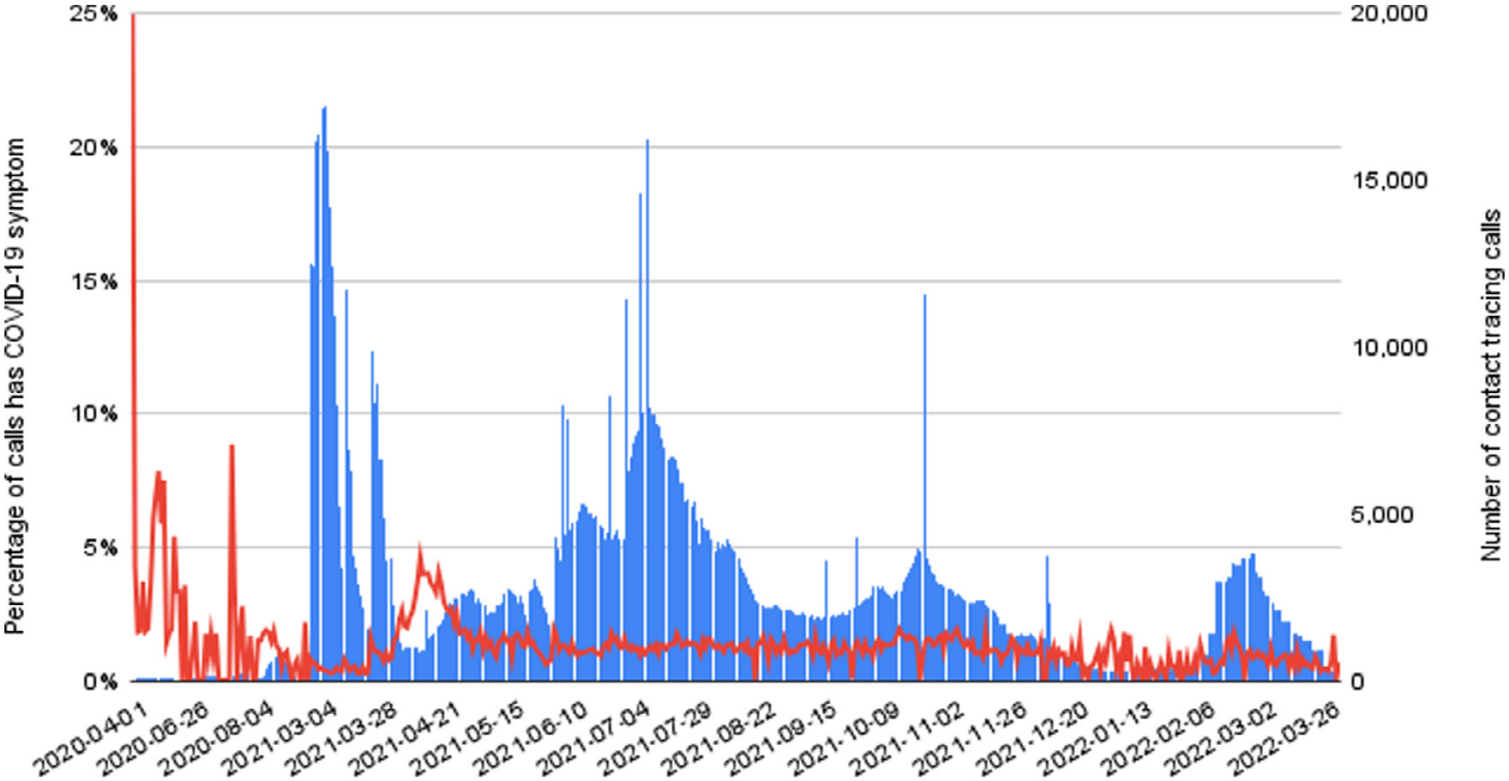
The blue line is the number of daily contact tracing automated calls, and corresponds to the right-hand side Y axis. The red line is the percentage of automated contact tracing call respondents with confirmed symptoms, and corresponds to the left-hand side Y axis between April 2020 and March 2022. Source: generated from the Hotline-115 system logs.

Between January 2016 and March 2022, Hotline-115 received an average of 592 incoming public calls each day, peaking at over 18,000 incoming calls per day from the public (Figure 2), and it sent out an average of 2,654 automated contact tracing calls per day between April 2020 and March 2022, 1.3% of respondents from the contact tracing calls met the COVID-19 symptoms definition (Figure 3).

## Discussion

Health workforce shortage is a global problem, but LMICs face even higher human resources constraints. In Cambodia, the CCDC has significantly less personnel and financial resources than its peers in developed countries and the staff would simply not be able to handle the volume of public engagement that was required to respond to the COVID-19 pandemic, if it was not for the ability to offload a significant part of the engagement to the Hotline-115 tool. Notably, only 1.3% of the people contacted by the contact tracing calls reported experiencing COVID-19 symptoms which required escalation to the contact tracer teams for human follow-up calls. In other words, 98.7% of all automated contact tracing calls required no human follow up - a huge saving in human resources, which resulted in the country being able to conduct a well-managed containment of the epidemic, even with the limited resources available.

The Hotline 115 system played an important role in CCDC's response and management of the COVID-19 pandemic, which potentially saved numerous human lives in Cambodia and mitigated collapse of its fragile labour-intensive economy. While the new National Hotline-115 system represents a leap forward, some challenges still remain. For example, the contact tracing process requires a manual step of exporting contact lists from the WHO Go.Data system and importing them to the Hotline-115 system, and further, the contact tracing results are not synchronised back to the Go.Data system.

The objective of public participation requires that any technology solution must meet inclusive design principles, and strive to lower the barriers to access for the population nationwide. In the case of the public reporting for any LMIC one needs to consider the adult literacy rates. In Cambodia for example, 19% of adults cannot read or write,^8^ hence, a voice based option was designed to accomodate the needs of the entire population. Furthermore, the Hotline-115 was designed for basic mobile phone users, not requiring Internet access or digital literacy.

Importantly, a technology solution is not sufficient if not fully deployed and widely adopted. To deploy Hotline-115 at the scale required for it to be impactful, it was essential for all stakeholders to be on board. In the case of Cambodia, CCDC provided the vision and the direction that enabled the necessary collaboration with other government agencies, the WHO, development partners, and the telecommunication operators. This resulted in successful deployment of the system, and minimised the disruption from the COVID-19 pandemic in Cambodia as much as possible.

## The way forward

The time to start working towards preparedness for the next pandemic is now. The COVID-19 pandemic stress test on the Hotline-115 demonstrated the utility of an ongoing, inclusive system for public participation in disease detection and response. We encourage leaders in other LMICs to invest in participatory surveillance through Hotlines or other methods that are inclusive by allowing participation with widely available technologies. The ability to scale with limited resources which are a fraction of the savings gained in human resources, time and money.

## Contributors

CL: writing - original draft, conceptualisation, methodology, project administration

SS: methodology, project administration, supervision

KC: conceptualisation, software, data curation, formal analysis

DL: writing - review and editing

VS: software

ND: supervision

SL: conceptualisation, methodology, supervision

MS: conceptualisation, methodology, funding acquisition.

## Declaration of interests

Divon Mordechai Lan reports a PhD scholarship from the University of Adelaide. The rest of the authors declare that they have no conflicts of interest.

## References

[1] Oppenheim B, Gallivan M, Madhav NK (2019). Assessing global preparedness for the next pandemic: Development and application of an Epidemic Preparedness Index. BMJ Glob Health.

[2] Lee L, Mukhi S, Bancej C. (2021). Crowdsourced disease surveillance success story: the FluWatchers program. Can Commun Dis Rep.

[3] Wójcik OP, Brownstein JS, Chunara R, Johansson MA. (2014). Public health for the people: participatory infectious disease surveillance in the digital age. Emerg Themes Epidemiol.

[4] EpiHack Phnom Penh. https://endingpandemics.org/projects/epihack/epihack-phnom-penh/. Accessed 22 July 2022.

[5] Cheney C. This nonprofit is helping communities stop pandemics in their tracks. Devex. 2021; published online May 17.https://www.devex.com/news/this-nonprofit-is-helping-communities-stop-pandemics-in-their-tracks-99890. Accessed 21 April 2022.

[6] Cambodia - 1 new outbreak of highly pathogenic avian influenza H5N1 in backyard birds, Svay Rieng Province (OIE, January 31, 2017). FluTrackers News and Information.https://flutrackers.com/forum/forum/cambodia/cambodia-h5n1-tracking/769442-cambodia-1-new-outbreak-of-highly-pathogenic-avian-influenza-h5n1-in-backyard-birds-svay-rieng-province-oie-january-31-2017. Accessed 21 April 2022.

[7] Cambodia COVID-19 statistics reported daily on the official CCDC Facebook page. https://www.facebook.com/cdcmohcam. Accessed 17 August 2022.

[8] Literacy rate, adult total (% of people ages 15 and above) - Cambodia. https://data.worldbank.org/indicator/SE.ADT.LITR.ZS?locations=KH. Accessed 21 April 2022.

